# LPS promotes the progression of sepsis by activation of lncRNA HULC/miR-204-5p/TRPM7 network in HUVECs

**DOI:** 10.1042/BSR20200740

**Published:** 2020-06-15

**Authors:** Xinghai Chen, Debiao Song

**Affiliations:** Department of Emergency and Critical Medicine, The Second Hospital of Jilin University, Changchun, Jilin 130041, China

**Keywords:** HULC, LPS, miR-204-5p, sepsis, TRPM7

## Abstract

Sepsis is a systemic inflammatory response syndrome caused by infection. Lipopolysaccharide (LPS) has been reported to induce inflammatory responses, and long non-coding RNA highly up-regulated in liver cancer (HULC) expression was associated with the progression of sepsis. But the role and underlying mechanism of HULC in LPS-induced sepsis remain unclear. Cell viability and apoptosis were measured by methyl thiazolyl tetrazolium (MTT) and flow cytometry assays, respectively. The levels of apoptosis-related proteins, inflammatory cytokines and transient receptor potential melastatin7 (TRPM7) were detected by western blot. Reactive oxygen species (ROS), superoxide dismutase (SOD) and malondialdehyde (MDA) levels were detected by dichloro-dihydro-fluorescein diacetate (DCFH-DA) method using commercial kit. HULC, microRNA-204-5p (miR-204-5p) and TRPM7 expressions in serum of sepsis patients and human umbilical vein endothelial cells (HUVECs) were examined by quantitative real-time polymerase chain reaction (qRT-PCR). Dual-luciferase reporter and RNA immunoprecipitation (RIP) assays were used to confirm the interaction between HULC and miR-204-5p, miR-204-5p and TRPM7. LPS stimulation restrained cell viability and facilitated apoptosis, inflammatory injury and oxidative stress in HUVECs. HULC and TRPM7 were increased and accompanied with decreased miR-204-5p expression in serum of sepsis patients. A significant negative correlation between miR-204-5p and HULC or TRPM7 was observed, and there was a positive relationship between expressions of HULC and TRPM7. Importantly, LPS inhibited the cell viability and induced apoptosis, inflammatory injury and oxidative stress of HUVECs by up-regulating the expressions of HULC and TRPM7, and down-modulating miR-204-5p expression. Mechanically, HULC positively regulated TRPM7 expression by sponging miR-204-5p in HUVECs. LPS impaired cell viability, and promoted cell apoptosis, inflammatory response and oxidative stress in HUVECs by regulating HULC/miR-204-5p/TRPM7 axis.

## Introduction

Sepsis is a systemic inflammatory disease caused by severe trauma, burns and postoperative infections. It develops rapidly and can lead to multiple organ failure [1]. It is estimated that in 2017, the percentage of ICU admissions due to sepsis was approximately 25%, and the global mortality rate was more than 50% [2]. Lipopolysaccharide (LPS), which is made up of lipids and polysaccharides, is also an endotoxin that is released from bacterial membranes and binds to receptors on the surface of endothelial cells, thereby acting as a toxic agent that causes severe inflammation [3]. LPS has been reported to induce sepsis by regulating the growth of endothelial cells, inflammatory factors and oxidative stress factors [4,5]. However, the exact mechanism by which LPS affects endothelial cell activity and induces cellular inflammation is still not fully understood.

Recently, the abnormally expressed long noncoding RNAs (lncRNAs) have been found in many sepsis cell models. For instance, lnc-IL7R was markedly enhanced in LPS-stimulated cells, and it could alleviate the LPS-induced inflammatory response [6]. LncRNA NEAT1 could regulate the LPS-induced oxidative stress in the heart [7]. LncRNA highly up-regulated in liver cancer (HULC) has been identified as an oncogene to modulate the development of many human cancers [8,9]; it promoted the growth and metastasis of tumor cells and tumorigenesis. In addition, Wang et al. demonstrated that HULC could be up-regulated under the stimulation of inflammatory factors and oxidative stress factors, thus promoting the migration and invasion of cholangiocyte [10]. This suggested that HULC played a pivotal role in inflammatory response and oxidative stress. Besides, microRNA-204-5p (miR-204-5p) has been shown to inhibit the inflammatory process of microglia cells caused by LPS [11]. In LPS-stimulated macrophages, the decreased expression of miR-204-5p was accompanied by the production of inflammatory cytokines [12]. Since sepsis induces inflammation and oxidative stress [13], it is necessary to explore the role and correlation between HULC and miR-204-5p in LPS-induced sepsis.

Transient receptor potential melastatin7 (TRPM7) is the main Ca^2+^-permeable channel and can activate fibroblasts [14]. Inhibition of TRPM7 could repress calcium influx and LPS-stimulated endothelial cell migration [15]. Additionally, Liu et al. found that TRPM7 was enormously increased in serum of sepsis patients [16], suggesting that TRPM7 might be implicated in the development of sepsis.

In the present research, we first used LPS to treat human umbilical vein endothelial cells (HUVECs) to establish an inflammatory HUVECs model. Next, we measured HULC expression in serum of sepsis patients and LPS-stimulated HUVECs, and investigated the potential mechanisms mediated by HULC in LPS-treated HUVECs.

## Materials and methods

### Blood samples, cell culture and LPS treatment

Blood samples were collected from 56 sepsis patients at The Second Hospital of Jilin University from January 2017 to May 2018, and 56 healthy volunteers were recruited. All sepsis patients and healthy volunteers completed informed consent forms. The serums were obtained by centrifuging the blood samples of sepsis patients and healthy volunteers at 3000 × *g* for 15 min and stored in an ultra-cold refrigerator at −80°C. This research was approved by the Ethics Committee of The Second Hospital of Jilin University.

HUVECs were purchased from American Tissue Culture Collection (ATCC, Manassas, VA, U.S.A.) and maintained in Dulbecco's modified Eagle's medium (DMEM, Thermo Fisher Scientific, Waltham, MA, U.S.A.) with 10% fetal bovine serum (FBS, Thermo Fisher Scientific) in an atmosphere of 5% CO_2_ at 37°C.

For LPS induction, HUVECs were inoculated in 6- or 96-well plates and incubated for 12 h. LPS (10 μg/ml) or saline (control) were treated HUVECs for 48 h, then cells were harvested for analysis of cell viability and apoptosis or transfected for further analysis.

### Cell viability and apoptosis assays

After HUVECs were inoculated for 12 h at 96-well plates, the cells were treated with LPS or saline for 48 h. 10 μl of methyl thiazolyl tetrazolium (MTT, Promega, Madison, WI, U.S.A.) was added to each well and incubated for another 2 h, and the absorbance at 490 nm was assessed by a microplate reader.

Flow cytometry assay was performed by using Annexin V-fluorescein isothiocynate (FITC)/propidium iodide (PI) apoptosis detection kit (Biosea Biotechnology, Beijing, China). HUVECs were transfected or treated with LPS for 48 h, cells were harvested, and treated with 10 μl Annexin V-FITC for 20 min, and then 10 μl PI was added to the cells for 20 min in the dark. Finally, cell apoptosis was analyzed by a flow cytometer (BD Biosciences, Franklin Lake, NJ, U.S.A.).

### Western blot assay

After extracting the proteins from HUVECs with RIPA (Thermo Fisher Scientific), they were first boiled at 98°C for 5 min to denature. The samples were separated and then transferred to polyvinylidene difluoride (PVDF, Millipore, Bedford, MA, U.S.A.) membranes. The membranes were blocked in 5% non-fat dry milk for 2 h, and incubated with primary antibody of B-cell lymphoma-2 (Bcl-2, 1:1000, ab32124, Abcam, Cambridge, MA, U.S.A.), Bcl-2-associated X (Bax, 1:2000, ab182733, Abcam), cleaved-caspase3 (1:500, ab32042, Abcam), tumor necrosis factor-α (TNFα, 1:1000, ab6671, Abcam), IL-6 (1:1000, 12912S, Cell Signaling Technology, Shanghai, China), IL-8 (1:1000, ab110727, Abcam), IL-1β (1:1000, 12703S, Cell Signaling Technology), TRPM7 (1:1000, ab109438, Abcam) or glyceraldehyde-3-phosphate dehydrogenase (GAPDH, 1:3000, ab8245, Abcam) at 4°C overnight. The membranes were washed and incubated with secondary antibodies for 1 h, and detected by an odyssey infrared imaging system (LI-COR Biosciences, Lincoln, NE, U.S.A.).

### Detection of reactive oxygen species, superoxide dismutase and malondialdehyde

Dichloro-dihydro-fluorescein diacetate (DCFH-DA, Beyotime, Shanghai, China) method was used to measure the level of reactive oxygen species (ROS) in HUVECs according to the product instruction, the transfected or LPS-treated HUVECs were incubated with 10 μM DCHF-DA for 15 min, then flow cytometry assay was performed to analyze the ROS production.

When HUVECs were treated with LPS or transfected for 48 h, cells were harvested and the levels of superoxide dismutase (SOD) and malondialdehyde (MDA) were examined by the commercial kit (Jiancheng Biotech, Nanjing, China) according to the manufacturer's instructions.

### Quantitative real-time polymerase chain reaction

The RNA in serum of sepsis patients or HUVECs was isolated by TRIZOL kit (Thermo Fisher Scientific). Then, the complementary DNA (cDNA) was synthesized by Prime Script RT Master Mix (Thermo Fisher Scientific), and a SYBR Green Master mix kit (Thermo Fisher Scientific) was used to perform quantitative real-time polymerase chain reaction (qRT-PCR) on the 7500 Real Time System (Applied Biosystems, Foster City, CA, U.S.A.). GAPDH and U6 were employed as the internal controls for HULC or TRPM7 and miR-204-5p, respectively, and the 2^−ΔΔCt^ method was used to calculate the relative expression. The following primers were synthesized by GenePharma (Shanghai, China). HULC, 5′-GCAAGCCAGGAAGAGTCGTC-3′ (F), 5′-GCTGTGCTTAGTTTATTGCCAGG-3′ (R). GAPDH, 5′-GCACCGTCAAGCTGAGAAC-3′ (F), 5′-TGGTGAAGACGCCAGTGGA-3′ (R). miR-204-5p, 5′-TTCCCTTTGTCATCCTATGCCT3-3′ (F), 5′-CAGTGCGTGTCGTGGAGT-3′ (R). U6, 5′-GTGCAGGGTCCGAGGT-3′ (F), 5′-CTCGCTTCGGCAGCACA-3′ (R). TRPM7, 5′-CACTTGGAAACTGGAACC-3′ (F), 5′-CGGTAGATGGCCTTCTACTG-3′ (R).

### Transfection

For knockdown of HULC, the small interference RNA (siRNA) against HULC (si-HULC) and control si-NC were synthesized by GenePharma. The mimic (miR-204-5p) and inhibitor (anti-miR-204-5p) of miR-204-5p and their respective controls (miR-NC and anti-miR-NC) were designed by GenePharma. For overexpression of TRPM7, its coding sequence (CDS) was cloned into the vector (Thermo Fisher Scientific). When the density of HUVECs reached 60–70%, transfection was performed using Lipofectamine 3000 (Invitrogen, Carlsbad, CA, U.S.A.).

### Dual-luciferase reporter assay

MiRcode and StarBase v2.0 were employed to predict the interaction among HULC, miR-204-5p and TRPM7. Wild type (WT) sequences of HULC containing miR-204-5p binding sites or mutant (MUT) sequences without binding sites were inserted into the pmirGLO vector (Promega) to form HULC WT and HULC MUT report plasmids. TRPM7 3′ UTR WT and TRPM7 3′ UTR MUT report plasmids were constructed in the same way. Then, the report plasmids were co-transfected with miR-204-5p or miR-NC into HUVECs using Lipofectamine 3000 (Invitrogen), respectively. 24 h later, a dual-luciferase reporter assay kit (Promega) was used to assess the luciferase activity.

### RNA immunoprecipitation assay

An EZ-Magna RIP Kit (Millipore) was used to analyze the relationship between HULC and miR-204-5p in RNA immunoprecipitation (RIP) assay. In brief, HUVECs were lysed in lysis buffer, then, incubated with magnetic beads conjugated to anti-Ago2, and anti-IgG group was used as the negative control. The co-precipitated RNAs from the beads were extracted and examined by qRT-PCR.

### Statistical analysis

Data were all appeared as the mean ± standard deviation (SD) from three independent experiments. Statistical evaluation was analyzed by using student's *t*-test between two groups or one-way analysis of variance (ANOVA) among multiple groups. *P  *<0.05 was deemed notably different.

## Results

### LPS reduced cell viability and induced apoptosis, inflammatory injury and oxidative stress in HUVECs

**Figure 1:**
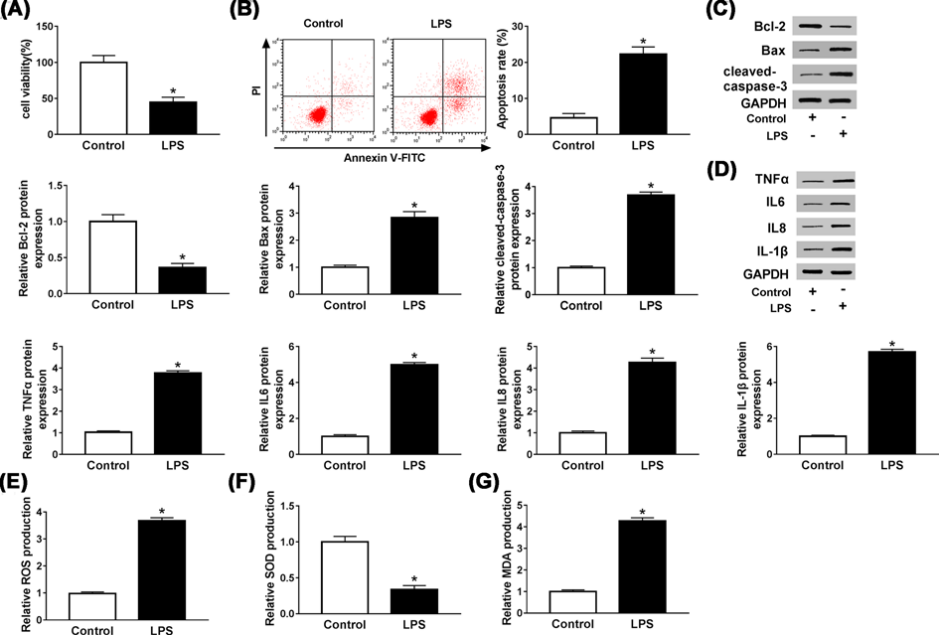
LPS reduced cell viability and induced apoptosis, inflammatory injury and oxidative stress in HUVECs (**A,B**) MTT and flow cytometry assays were used to determine the viability and apoptosis of LPS-induced HUVECs, respectively. (**C,D**) The levels of apoptosis-related proteins Bcl-2, Bax, cleaved-caspase3 and inflammation-related cytokines TNFα, IL-6, IL-8 and IL-1β in LPS-treated HUVECs were detected by western blot. (**E–G**) The productions of ROS, SOD and MDA were measured by DCFH-DA method and a commercial kit. *P<0.05.

To evaluate the effect of LPS (10 μg/ml) on the properties of HUVECs, MTT and flow cytometry assays were performed to detect viability and apoptosis of HUVECs, respectively. The results showed that LPS repressed cell viability (*P* = 0.0013, Figure 1A) and increased the rate of apoptosis (*P* = 0.0002, Figure 1B). Meanwhile, after HUVECs were treated with LPS, western blot detected that the expression levels of Bax and cleaved-caspase3 were increased (*P* = 0.0002 and 0.0002), while Bcl-2 was decreased (*P* = 0.0006, Figure 1C), which further indicated that LPS could promote apoptosis of HUVECs. Then we measured the expressions of inflammatory cytokines by western blot. As shown in Figure 1D, the protein levels of TNFα, IL-6, IL-8 and IL-1β were significantly elevated in LPS-induced HUVECs (*P*<0.0001, *P*<0.0001, *P*<0.0001 and *P*<0.0001), suggesting that LPS treatment could trigger inflammation. Furthermore, to explore the effects of LPS on cellular redox status in HUVECs, we evaluated the levels of ROS, SOD and MDA. As displayed in Figure 1E–G, ROS production and MDA production were distinctly increased by LPS stimulation (*P*<0.0001 and *P*<0.0001), while SOD production was substantially dwindled (*P* = 0.0003). These results indicated that LPS suppressed cell viability, induced apoptosis, inflammatory response and oxidative stress in HUVECs.

### HULC was up-regulated in serum of sepsis patients and in LPS-induced HUVECs

**Figure 2:**
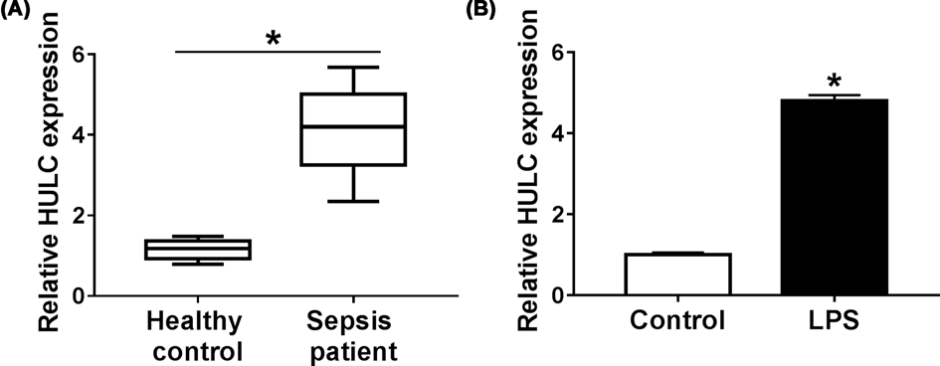
HULC was up-regulated in serum of sepsis patients and in LPS-induced HUVECs (**A**) HULC expression in serum of sepsis patients compared with healthy controls was measured by qRT-PCR. (**B**) HULC expression was examined by qRT-PCR in HUVECs treated with LPS. *P<0.05.

We then performed qRT-PCR analysis to measure HULC expression in serum of sepsis patients. Compared with the healthy controls, HULC expression was enhanced in serum of sepsis patients (*P*<0.0002, Figure 2A). Based on the median of HULC expression, sepsis patients were divided into high HULC expression group and low HULC expression group. The analysis of sepsis patients’ clinical features revealed that high HULC expression was associated with the severity of sepsis (Table 1). To investigate the effect of LPS on HULC expression, HUVECs were treated with LPS (10 μg/ml) for 48 h, and the control group was not given any treatment. As shown in Figure 2B, LPS could increase HULC expression in HUVECs (*P* = 0.0002). These results implied that LPS might inhibit the progression of HUVECs by up-regulating HULC expression.

**Table 1:** Association of HULC expressions with the baseline clinical features of sepsis patients (*n* = 30)

Parameter	Case	HULC expression		*P* value^*^
		Low (*n* = 15)	High (*n* = 15)	
Age (years)				0.7048
≤55	19	9	10	
>55	11	6	5	
Gender				0.2690
Male	17	10	7	
Female	13	5	8	
APACHE II score				0.0053^†^
≤17.15	9	8	1	
>17.15	21	7	14	
SOFA score				0.0253^†^
≤7.64	12	9	3	
>7.64	18	6	12	

APACHE, acute physiology and chronic health evaluation; SOFA, sequential organ failure assessment. ^*^Chi-square test. ^†^*P* < 0.05.

### HULC knockdown could alleviate LPS-induced injury of HUVECs

**Figure 3:**
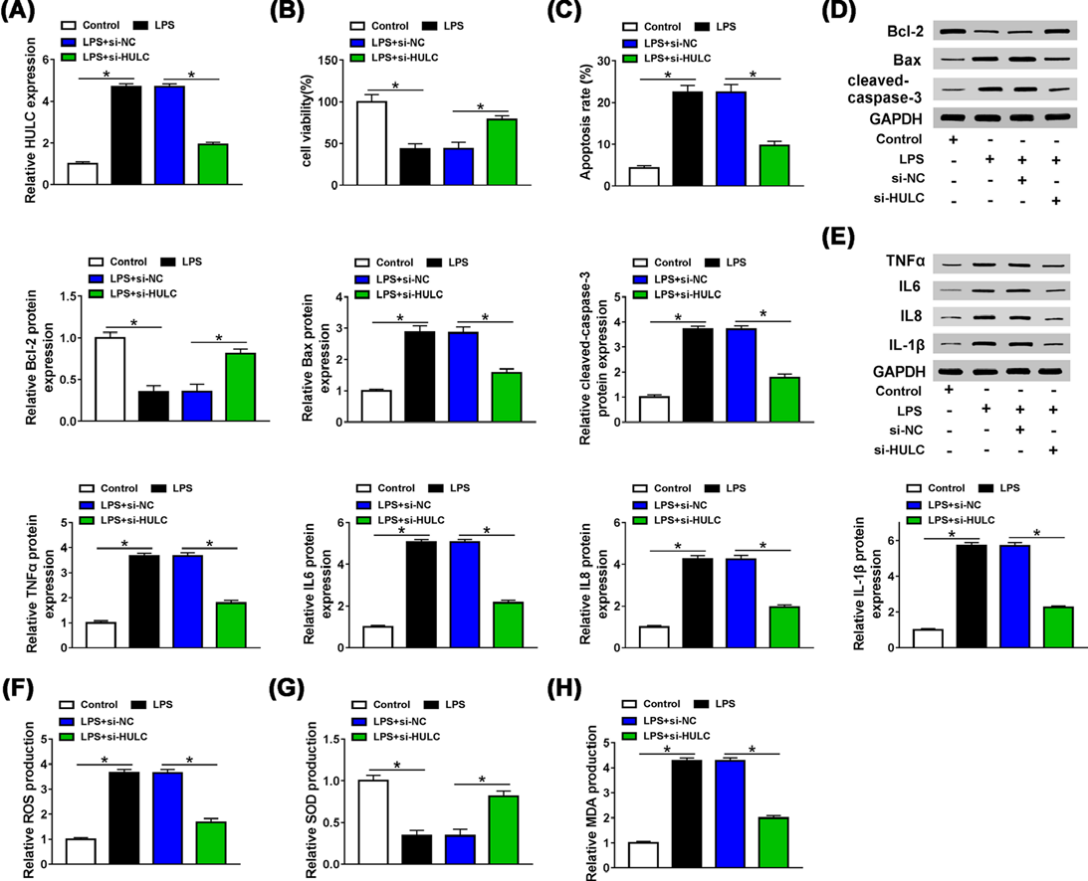
HULC knockdown could rescue the inhibitory effect of LPS on progression of HUVECs HUVECs were treated with LPS for 48 h, and cells in control group were not given any treatment and after LPS-treated HUVECs were transfected with si-HULC or si-NC. (**A**) HULC expression was measured by qRT-PCR. (**B,C**) MTT and Flow cytometry assays were performed to detect cell viability and apoptosis, respectively. (**D,E**) The levels of Bcl-2, Bax, cleaved-caspase3, TNFα, IL-6, IL-8 and IL-1β were determined by western blot. (**F–H**) ROS, SOD and MDA productions were examined by DCFH-DA method and a commercial kit. *P<0.05.

To figure out whether HULC affected the effect of LPS on HUVECs, HUVECs were separately divided into four groups, namely control, LPS, LPS + si-NC and LPS + si-HULC. The transfection efficiency was first examined by qRT-PCR. Three siRNAs against HULC (si-HULC#1, si-HULC#2 and si-HULC#3) were used to silence HULC in LPS induced HUVECs, and the knockdown efficiency of si-HULC#1 was most significant (data not shown), so we used si-HULC#1 as si-HULC for subsequent experiments. LPS elevated HULC expression (*P*<0.0001), while this effect was reversed by interfering HULC (*P*<0.0001, Figure 3A). Besides, suppression of HULC could partially alleviate the inhibitory ability of LPS on cell activity (*P* = 0.0012, Figure 3B) and the promoting effect of LPS on apoptosis (*P*<0.0001, Figure 3C). LPS increased the levels of Bax (*P*<0.0001), cleaved-caspase3 (*P*<0.0001) and declined Bcl-2 (*P*<0.0001) expression, and these effects were weakened by transfection of si-HULC (*P*<0.0001, *P*<0.0001, *P* = 0.0002, Figure 3D). As presented in Figure 3E, the facilitation effects of LPS on the protein levels of TNFα, IL-6, IL-8 and IL-1β were reversed by silencing HULC (*P*<0.0001, *P*<0.0001, *P*<0.0001, *P*<0.0001). Simultaneously, LPS increased the productions of ROS (*P*<0.0001) and MDA (*P*<0.0001) and reduced the production of SOD (*P*<0.0001), which could be overturned by inhibiting HULC expression (*P*<0.0001, *P*<0.0001, *P* = 0.0001, Figure 3F–H). The above results signified that LPS promoted the progression of sepsis by regulating HULC expression.

### HULC functioned as a sponge for miR-204-5p

**Figure 4:**
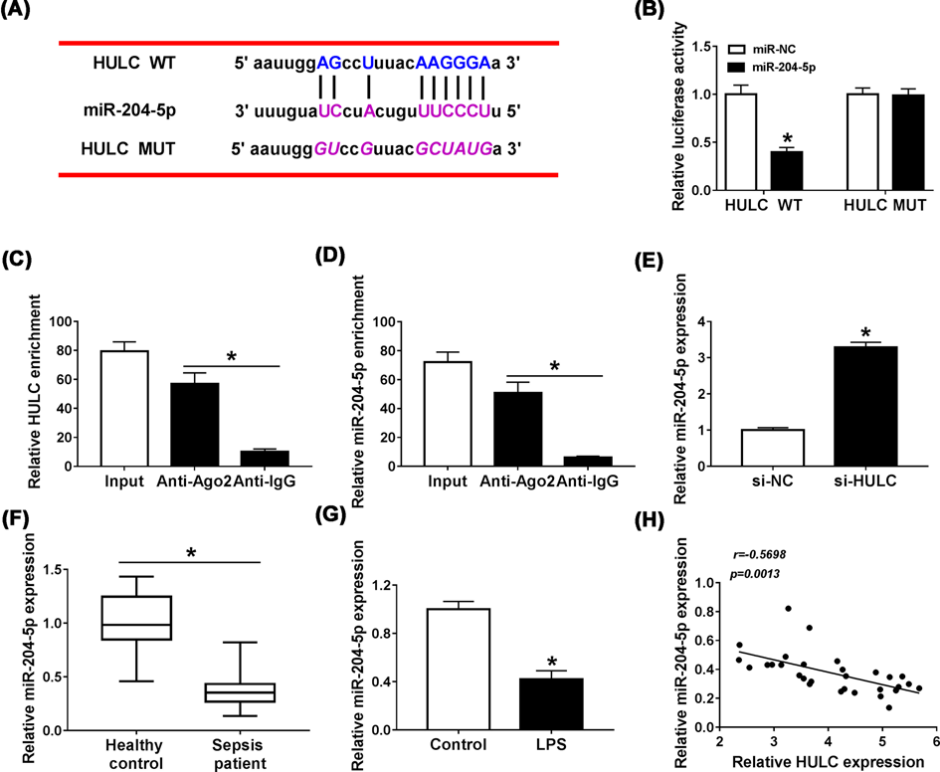
HULC functions as a sponge for miR-204-5p (**A**) MiRcode software was employed to predict the target miRNAs of HULC. (**B**) After HUVECs were co-transfected with HULC WT or HULC MUT and miR-204-5p or miR-NC, the luciferase activities were analyzed using dual-luciferase reporter assay. (**C,D**) RIP assay was carried out in HUVECs, and the levels of HULC and miR-204-5p were measured by qRT-PCR. (**E**) The expression of miR-204-5p in HUVECs transfected with si-HULC or si-NC was examined by qRT-PCR. (**F**) MiR-204-5p expression in serum of sepsis patients compared with healthy controls was measured by qRT-PCR. (**G**) MiR-204-5p expression was determined by qRT-PCR in HUVECs treated with LPS. (**H**) The correlation between HULC expression and miR-204-5p expression was assessed by Pearson correlation coefficient. *P<0.05.

LncRNAs are generally served as competitive endogenous RNAs (ceRNAs) of miRNAs to regulate the activity of miRNA and thus play a biological role [17]. We used MiRcode biological software to analyze the target miRNAs of HULC and found that there were binding sites for miR-204-5p in HULC (Figure 4A). Dual-luciferase reporter and RIP assays were performed to verify the interaction between them. As appeared in Figure 4B, miR-204-5p obviously decreased the luciferase activity of HULC WT (*P*<0.0001), but did not significantly change the luciferase activity of HULC MUT (*P* = 0.97). RIP assay results showed that Ago2 antibody strikingly enriched the expression levels of miR-204-5p (*P* = 0.0002) and HULC (*P* = 0.0002) compared with IgG antibody (Figure 4C,D). And HULC knockdown enhanced the expression of miR-204-5p in HUVECs (*P*<0.0001, Figure 4E). Subsequently, we examined miR-204-5p expression by qRT-PCR in serum of sepsis patients and found that miR-204-5p expression was down-regulated in serum of sepsis patients in contrast with the healthy controls (*P*<0.0001, Figure 4F). Contrary to the promotion effect of LPS on HULC expression, miR-204-5p was decreased in LPS-treated HUVECs (*P* = 0.0005, Figure 4G). Not surprisingly, HULC expression was negatively correlated with miR-204-5p expression in serum of sepsis patients (Figure 4H). These findings supported that miR-204-5p was a target miRNA of HULC.

### Suppression of HULC increased cell viability and restrained apoptosis, inflammatory response and oxidative stress by targeting miR-204-5p in LPS-induced HUVECs

**Figure 5:**
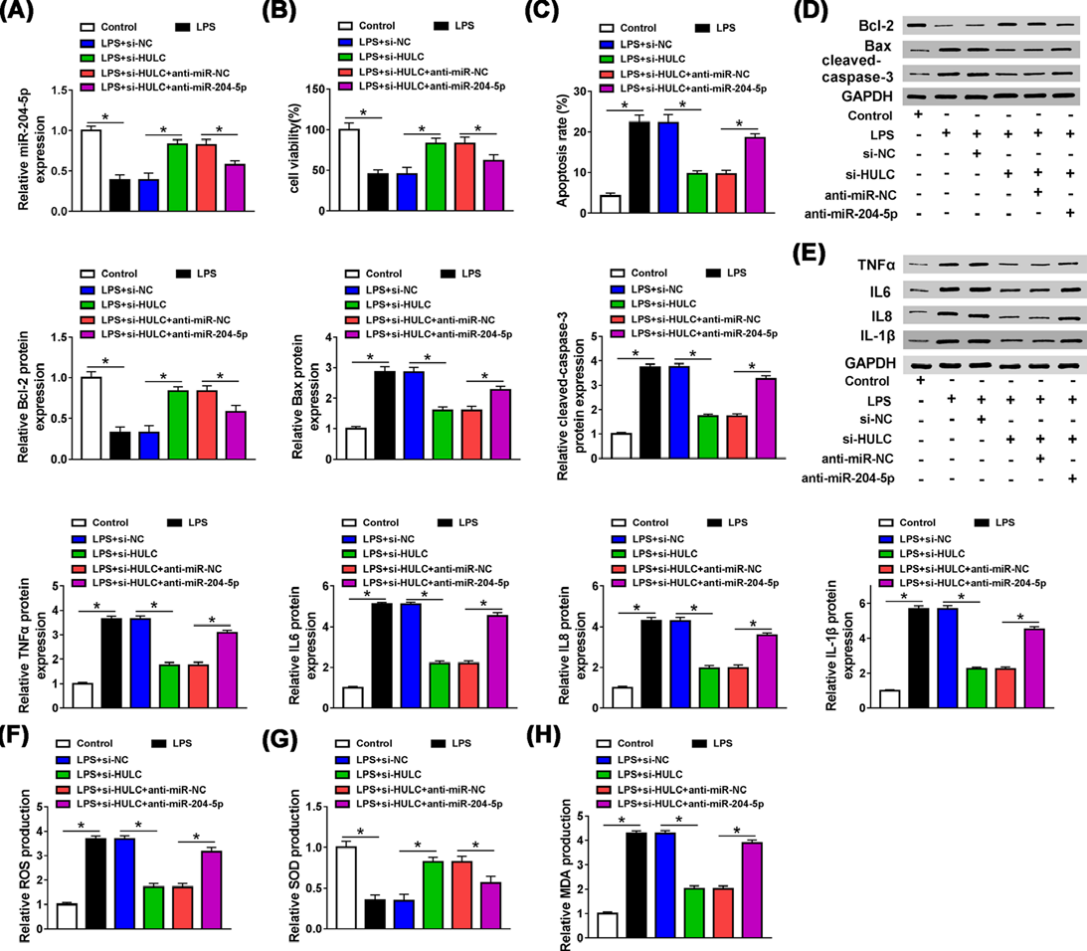
Suppression of HULC increased cell viability and restrained apoptosis, inflammatory response and oxidative stress by targeting miR-204-5p in LPS-induced HUVECs HUVECs were treated with LPS for 48 h, and cells in control group were not given any treatment, and then LPS-treated HUVECs were transfected with si-NC, si-HULC, si-HULC + anti-miR-NC or si-HULC + anti-miR-204-5p. (**A**) MiR-204-5p expression was detected by qRT-PCR. (**B,C**) The cell viability and apoptosis were evaluated by using MTT and Flow cytometry assays, respectively. (**D,E**) Western blot assay was performed to examine the protein levels of Bcl-2, Bax, cleaved-caspase3, TNFα, IL-6, IL-8 and IL-1β. (**F**–**H**) ROS, SOD and MDA productions were determined by using DCFH-DA method and a commercial kit. *P<0.05.

We further explored whether HULC was involved in the progression of HUVECs by regulating miR-204-5p expression. First transfection of si-HULC could attenuate the inhibition of LPS on miR-204-5p expression (*P*<0.0001), and interference with miR-204-5p counteracted the promoting effect of si-HULC on miR-204-5p expression (*P* = 0.0043, Figure 5A). MTT and Flow cytometry assays demonstrated that HULC knockdown induced cell viability (*P* = 0.0012) and hampered apoptosis (*P*<0.0001) in LPS-stimulated HUVECs, and these effects were abolished by co-transfection with anti-miR-204-5p (*P* = 0.0324, *P*<0.0001, Figure 5B,C). In addition, the effects of HULC deficiency on levels of Bax, Bcl-2 and cleaved-caspase3 in LPS-treated HUVECs could also be abolished by interfering with miR-204-5p (*P* = 0.0006, *P*<0.0001, *P* = 0.0116, Figure 5D). Similarly, the inhibitory effects of si-HULC on levels of TNFα, IL-6, IL-8 and IL-1β in LPS-treated HUVECs were abated by co-transfection with anti-miR-204-5p (*P*<0.0001, *P*<0.0001, *P*<0.0001, *P*<0.0001, Figure 5E). Meanwhile, knockdown of HULC repressed the productions of ROS (*P*<0.0001) and MDA (*P*<0.0001), induced SOD (*P* = 0.0001) production, as expected, silencing miR-204-5p could neutralized the effect of si-HULC on these oxidative stress indicators in LPS-treated HUVECs (*P*<0.0001, *P*<0.0001, *P* = 0.01, Figure 5F–H). These observations revealed that HULC inhibited the progression of HUVECs by targeting miR-204-5p in LPS-treated HUVECs.

### The 3′ UTR of TRPM7 was directly targeted by miR-204-5p

**Figure 6:**
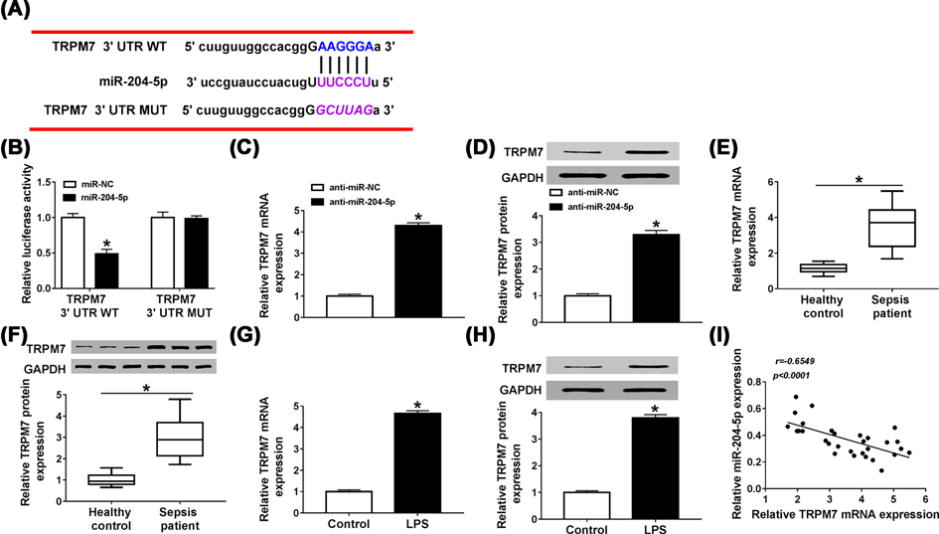
The 3′ UTR of TRPM7 was directly targeted by miR-204-5p (**A**) StarBase v2.0 was predicted that there were binding sites between miR-204-5p and TRPM7 3′ UTR. (**B**) Dual-luciferase reporter assay was performed to confirm the correlation between miR-204-5p and TRPM7, and the luciferase activity of transfected HUVECs was detected. (**C,D**) The mRNA and protein levels of TRPM7 in HUVECs transfected with anti-miR-NC or anti-miR-204-5p were assessed by qRT-PCR and western blot, respectively. (**E,F**) TRPM7 mRNA and protein levels in serum of sepsis patients and healthy controls were examined by qRT-PCR and western blot, respectively. (**G,H**) After HUVECs were treated with LPS or without any treatment, TRPM7 mRNA and protein levels were determined by qRT-PCR and western blot, respectively. (**I**) The correlation between the expressions of miR-204-5p and TRPM7 was analyzed using Pearson correlation coefficient. *P<0.05.

The next we searched the target mRNAs of miR-204-5p by StarBase v2.0 and found that there were binding sites between miR-204-5p and TRPM7 3′ UTR (Figure 6A). Then dual-luciferase reporter assay was performed to verify this prediction and the results showed that the luciferase activity of TRPM7 3′ UTR WT was prominently reduced by transfection with miR-204-5p (*P*<0.0001), while there was no significant difference in luciferase activity of TRPM7 3′ UTR MUT (*P* = 0.9348, Figure 6B). After HUVECs were transfected with anti-miR-204-5p, the mRNA and protein levels of TRPM7 were up-regulated (*P*<0.0001, *P*<0.0001, Figure 6C,D). We then examined the expression of TRPM7 in serum of patients with sepsis. The data revealed that TRPM7 was strikingly fortified in mRNA and protein levels in serum of sepsis patients compared with that in healthy controls (*P*<0.0001, *P*<0.0001, Figure 6E,F). Besides, LPS could aggrandize TRPM7 expression at mRNA and protein levels (*P*<0.0001, *P*<0.0001, Figure 6G,H). A significant inverse correlation between miR-204-5p expression and TRPM7 expression in serum of sepsis patients was analyzed by Pearson correlation coefficient (*P*<0.0001, Figure 6I). These data demonstrated that miR-204-5p could directly target TRPM7 and negatively regulate its expression in HUVECs.

### MiR-204-5p promoted LPS-induced injury of HUVECs by down-regulating TRPM7 expression

**Figure 7:**
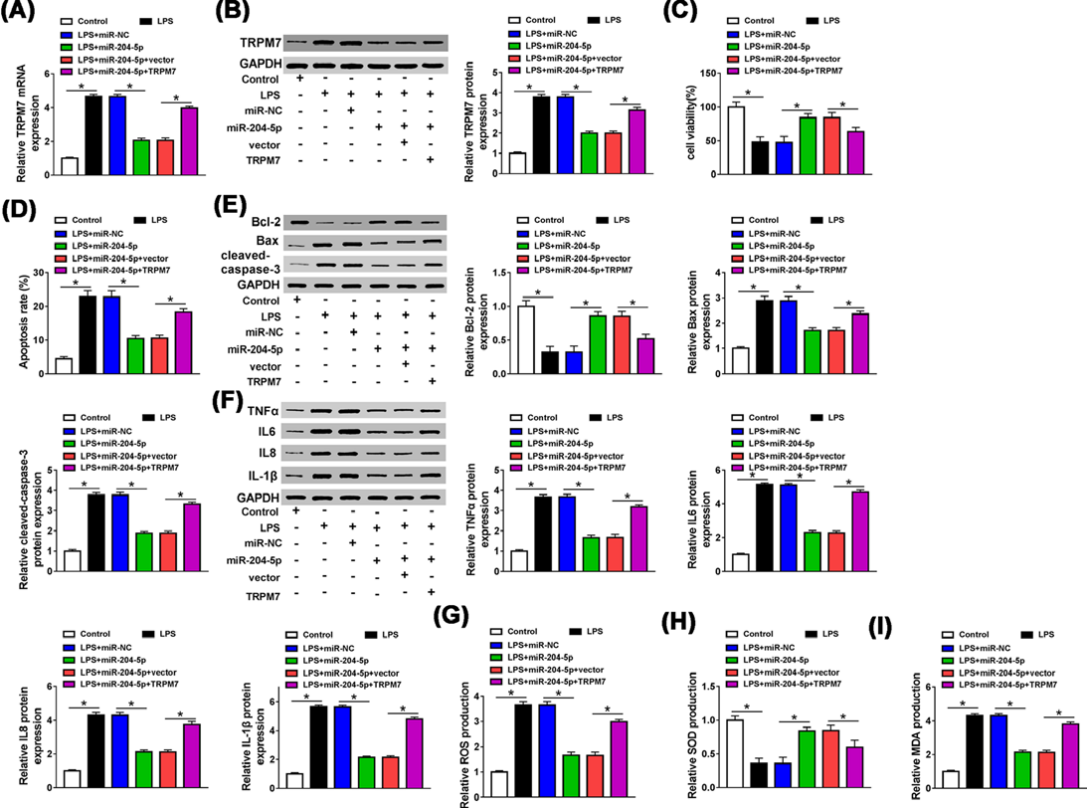
MiR-204-5p promoted the development of LPS-treated HUVECs by down-regulating TRPM7 expression After LPS-treated HUVECs were transfected with miR-NC, miR-204-5p, miR-204-5p + vector or miR-204-5p + TRPM7, and cells in control group were not given any treatment. (**A,B**) TRPM7 mRNA and protein levels were measured by qRT-PCR and western blot, respectively. (**C,D**) The viability and apoptosis of transfected HUVECs were assessed by MTT and Flow cytometry assays, respectively. (**E,F**) The protein levels of Bcl-2, Bax, cleaved-caspase3, TNFα, IL-6, IL-8 and IL-1β were detected using western blot assay. (**G–I**) The productions of ROS, SOD and MDA were assessed by DCFH-DA method and a commercial kit. *P<0.05.

In order to investigate the effect of TRPM7 in HUVECs, miR-204-5p and TRPM7 were co-transfected into LPS-treated HUVECs, and the transfection efficiency was measured by qRT-PCR and western blot assays. Overexpression of miR-204-5p reduced the mRNA and protein levels of TRPM7 in LPS-treated HUVECs (*P*<0.0001, *P*<0.0001), and co-transfection of TRPM7 reversed this effect (*P*<0.0001, *P*<0.0001, Figure 7A,B). Meanwhile, up-regulation of TRPM7 inverted the promotion effect of miR-204-5p on cell viability (*P* = 0.0417, Figure 7C) and the inhibition on apoptosis (*P* = 0.0001, Figure 7D) in LPS-treated HUVECs. In HUVECs, miR-204-5p weakened the reduction effect of LPS on Bcl-2 (*P* = 0.0002) level and the facilitation effects of LPS on levels of Bax (*P*<0.0001), cleaved-caspase3 (*P*<0.0001), TNFα (*P*<0.0001), IL-6 (*P*<0.0001), IL-8 (*P*<0.0001) and IL-1β (*P*<0.0001), however, these effects of miR-204-5p were rescued by overexpressing TRPM7 (*P* = 0.0016, *P* = 0.0007, *P*<0.0001, *P*<0.0001, *P*<0.0001, *P*<0.0001, *P*<0.0001, Figure 7E,F). In addition, transfection of miR-204-5p in LPS-treated HUVECs suppressed the productions of ROS (*P*<0.0001) and MDA (*P*<0.0001), augmented the production of SOD (*P*<0.0001). As expected, these changes were neutralized when TRPM7 was overexpressed (*P*<0.0001, *P* = 0.0247, *P*<0.0001, Figure 7G–I). Taken together, miR-204-5p regulated the development of LPS-treated HUVECs by targeting TRPM7.

### Knockdown of HULC down-regulated TRPM7 expression by serving as miR-204-5p sponge

**Figure 8:**
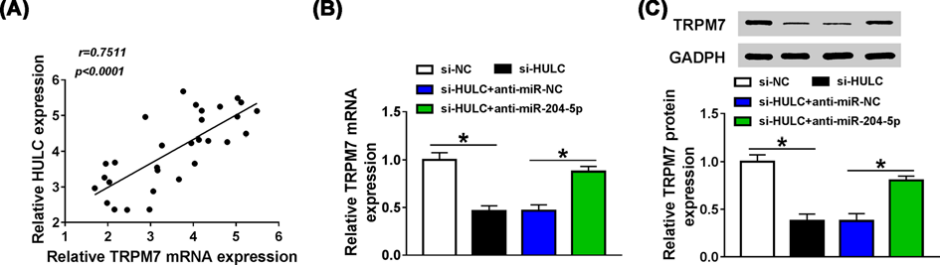
Knockdown of HULC down-regulated TRPM7 expression by serving as miR-204-5p sponge (**A**) Pearson correlation coefficient was used to analyze the correlation between HULC expression and TRPM7 expression in serum of sepsis patients. (**B,C**) After HUVECs were transfected with si-NC, si-HULC, si-HULC + anti-miR-NC or si-HULC + anti-miR-204-5p, the mRNA and protein levels of TRPM7 were determined by qRT-PCR and western blot, respectively. *P<0.05.

The ceRNA regulation network of lncRNA-miRNA-mRNA has been widely reported in Sepsis [18]. So we explore the relationship between HULC and TRPM7. Pearson correlation coefficient showed that HULC expression was positively associated with the expression of TRPM7 in serum of sepsis patients (Figure 8A). Meanwhile, when HULC was knocked down in HUVECs, the mRNA and protein levels of TRPM7 were decreased (*P*<0.0001, *P*<0.0001), and co-transfection with anti-miR-204-5p rescued the effect of si-HULC on TRPM7 expression (*P* = 0.0002, 0.0003, Figure 8B,C), indicating that HULC could act as a ceRNA of miR-204-5p to enhance TRPM7 expression in HUVECs.

## Discussion

The injury of HUVECs was considered as a leading feature of sepsis, and LPS-induced HUVECs have been widely used as an *in vitro* model of sepsis in many researches [19–21]. In the present study, we successfully established a model of LPS-induced inflammatory HUVECs, and the results showed that LPS retarded cell viability, induced apoptosis, inflammatory injury and oxidative stress in HUVECs. Our data indicated that HULC potentiated in sepsis patients and accompanied with decreased miR-204-5p expression and enhanced TRPM7 expression. Furthermore, LPS could elevate the levels of HULC and TRPM7, and restrain miR-204-5p expression in HUVECs. The recovery experiments proved that LPS induced the development of sepsis by regulating HULC/miR-204-5p/TRPM7 axis.

LPS is a cytotoxic agent that can induce inflammatory response and increase the expressions of inflammatory factors TNFα, IL-6, IL-8 and IL-1β, leading to sepsis [22]. LPS could also promote the apoptosis of inflammatory HUVECs by affecting the levels of apoptosis-related proteins Bcl-2, Bax and cleaved-caspase3 [23]. Moreover, Zhang et al. revealed that LPS could promote the productions of ROS and MDA, and hamper the production of SOD [24]. In accordance with these data, our results showed that LPS could impair cell viability and promote cell apoptosis by increasing the levels of Bax and cleaved-caspase3 and decreasing Bcl-2 level, In addition, LPS stimulation could increase the expressions of TNFα, IL-6, IL-8 and IL-1β to trigger inflammatory response, and up-modulate the productions of ROS and MDA as well as decline SOD production to induce oxidative stress response.

HULC plays an oncogene role in a variety of cancers [25,26], and a recent report revealed that increased HULC expression was related to LPS-induced inflammatory response and sepsis in endothelial cells [27]. Recently, the role of miR-204-5p in sepsis has been gradually explored, and it could target ANG-1 and be targeted by lncRNA NEAT1 to inhibit the damage of LPS on cells [28,29]. LncRNA generally functions as ceRNA of miRNA to regulate mRNA expression [30]. So, we wondered whether HULC and miR-204-5p were related in LPS-induced HUVECs. Coincidently, HULC could serve as a sponge for miR-204-5p, and their expression patterns were reversed in serum of sepsis patients, with HULC increase and miR-204-5p decrease. An evident inverse correlation between them was also found. In LPS-stimulated HUVECs, HULC was increased, and miR-204-5p was dwindled. Meanwhile, we found that anti-miR-204-5p could reverse the effects of si-HULC on cell viability, apoptosis, inflammatory response and oxidative stress in LPS-treated HUVECs. These results supported that LPS regulated the progression of HUVECs by regulating HULC/miR-204-5p axis.

TRPM7 ion channel has been reported to be a key protein in the regulation of inflammatory response during sepsis [31], and TRPM7 was also involved in LPS-induced transformation of endothelial cells into activated fibroblasts [32]. We predicted and confirmed that TRPM7 was a target of miR-204-5p. Not only that, HULC could actively modulate TRPM7 expression by sponging miR-204-5p. Similarly, LPS could promote TRPM7 expression in HUVECs. Functionally, TRPM7 overexpression could rescue the effect of miR-204-5p on the development of LPS-treated HUVECs.

However, it may be complicated for the regulatory mechanism of HULC in sepsis. Multiple miRNAs have been identified as targets of HULC in different diseases, such as miR-593 in hepatocellular carcinoma, miR-372 in liver cancer and so on [25,33]. Similarly, miR-204-5p also could bind to various genes, including ribonucleotide reductase M2 (RRM2), autophagy-related gene 3 (ATG3) and IL-6 receptor α (IL6R) [34–36]. In our study, we only illuminated the regulatory mechanism of LPS/HULC/miR-204-5p/TRPM7 axis in HUVECs. Whether HULC or miR-204-5p has other targets in sepsis still needs to be explored further. In addition, our study also had some limitations. As an ion channel, TRPM7 was closely related with intracellular signaling [37], yet we did not explore whether HULC/miR-204-5p/TRPM7 axis affected sepsis development via regulating some signaling pathways. Besides, we did not investigate the role of HULC/miR-204-5p/TRPM7 axis in sepsis *in vivo* models. These issues will be our concerns in the further study.

In conclusion, HULC and TRPM7 expression levels were up-regulated in sepsis patients and LPS-induced HUVECs, while miR-204-5p was down-regulated. HULC acted as miR-204-5p sponge to positively modulate TRPM7 expression in HUVECs. Importantly, LPS reduced cell viability, facilitated apoptosis, inflammatory injury and oxidative stress in HUVECs by increasing the levels of HULC and TRPM7 as well as decreasing miR-204-5p level. These observations revealed that the LPS/HULC/miR-204-5p/TRPM7 network might play a pivotal role in the process of inflammation and oxidative stress in HUVECs, which might provide potential therapeutic target for sepsis patients.

## Data Availability

Please contact the correspondence author for the data request.

## Abbreviations

Bax: Bcl-2-associated X
Bcl-2: B-cell lymphoma-2
DCFH-DA: dichloro-dihydro-fluorescein diacetate
FITC: fluorescein isothiocynate
GAPDH: glyceraldehyde-3-phosphate dehydrogenase
HULC: highly up-regulated in liver cancer
HUVEC: human umbilical vein endothelial cell
lncRNA: long noncoding RNA
LPS: lipopolysaccharide
MUT: mutant
PI: propidium iodide
qRT-PCR: quantitative real-time polymerase chain reaction
MTT: methyl thiazolyl tetrazolium
ROS: reactive oxygen species
siRNA: small interference RNA
SOD: superoxide dismutase
WT: wild type
